# Ionic Liquids 2014 and Selected Papers from ILMAT 2013: Highlighting the Ever-Growing Potential of Ionic Liquids

**DOI:** 10.3390/ijms151222815

**Published:** 2014-12-09

**Authors:** André Vioux, Andreas Taubert

**Affiliations:** 1 Institut Charles Gerhardt de Montpellier, UMR 5253 CNRS-UM2-ENSCM-UM1, Université Montpellier 2, Place Eugène Bataillon, Montpellier cc1701, France; 2 Institute of Chemistry, University of Potsdam, Karl-Liebknecht-Str. 24–25, Potsdam D-14476, Germany

## 1. Editorial

Ionic Liquids (ILs) are arguably among the most intensely researched areas in today’s natural sciences, especially the chemistry, physics, and materials sciences fields. The high promise of ILs for essentially all fields of science, engineering, and technology has also led to a sprouting of national and international meetings focusing on ILs and their use and application. Probably, the largest IL meeting is the Conference on Ionic Liquids (COIL) conference, now going into its 6^th^ edition, to take place in Korea in 2015.

In contrast to the rather large COIL Meetings, the Ionic Liquid-based Materials (ILMAT) conferences are somewhat of a Gordon Conference style meeting, focusing on materials chemistry using ILs as a materials design tools, hence the name of the conference. The ILMAT conference series was initiated in 2011 by Marie-Alexandra Néouze, then at the Technical University of Vienna, where in December 2011 about 50 participants gathered to discuss materials-related aspects of ionic liquids. As the interest in the topic continued to grow and as the ILMAT 2011 was a great success both on the scientific and collaborative levels, it was decided on the spot that a second ILMAT was to take place in 2013. André Vioux and colleagues from the Université de Montpellier agreed to become the organizers and again, the ILMAT conference was a resounding success. At ILMAT 2013, already over 80 participants eager to discuss advances in ionic liquid-based materials chemistry and physics participated. As a result of these two successful meetings, the ILMAT series will be continued. ILMAT 2015 is scheduled to take place in Potsdam in November 2015.

Before attending ILMAT 2015, however, we cordially invite you to browse the International Journal of Molecular Sciences special issue “Ionic Liquids 2014 and Selected Papers from ILMAT 2013”, which gathers highlights from the ILMAT 2013 meeting and articles that were contributed to this special issue in response to the open call for articles. The articles clearly show that ILs have not reached their peak potential yet. Rather, we are currently in the process of developing an understanding how the many interesting chemical and physical properties of each individual IL can be put to use in various fields. The special issue assembles some of these directions.

For example, right in the focus of ILMAT 2013, Kirchhöfer *et al.* [1] describe new separators for lithium ion batteries; the main outcome of the study is that the wetting of the membrane base materials by the ILs is a key aspect for proper functionality of the membrane. While hydrophobic membranes are poorly wet by hydrophobic ILs more hydrophilic ILs efficiently wet the membrane supports. Moreover, the study also shows that the membrane morphology is a key aspect for ensuring proper function. Further support for the high potential of these systems comes from Grande *et al.* [2], from the same laboratory, showing that indeed *N*-butyl-*N*-methylpyrrolidinium bis(trifluoromethanesulfonyl)imide is a viable component for high performance Li ion batteries.

A completely different focus, but still using polymers and ILs, is the basis of a study by Gallagher *et al.* [3]. These authors developed semi-interpenetrating networks based on polymerized ILs and poly(*N*-isopropylacrylamide) with an improved shrinking-swelling performance. The authors suggest that the resulting materials could be used in microfluidic controls. Alternatively, actuators on the basis of such materials could also be envisioned.

A further article by Martinelli [4] also focuses on materials synthesis using ILs, in this case silica is the material of interest. Silica-based ionic liquid composites have been made before [5] but the formation mechanism of IL/silica hybrid materials (silica ionogels) has still not been elucidated in detail and the kinetics of silica condensation have not been clarified completely. The article thus clearly contributes to a better understanding of IL-assisted (hybrid) material formation. This understanding should in turn then also enable a better a priori materials design, which is important for tuning ionogels for specific applications.

Isik *et al*. [6] review approaches on how the ability of ILs to dissolve carbohydrates enables the generation of new materials by chemical transformation of cellulose. This is certainly one of the most promising research alleys for materials development in the future: cellulose is among the most abundant raw materials on earth and there are numerous reactions in place for chemical modification of the cellulosic -OH groups. As a result, a multitude of different derivatives can be envisioned. These will then again provide access to materials for many different fields of application. Indeed, Liu *et al*. [7] demonstrate that ILs can be used to generate stationary phases for chiral separation. The cellulose base polymer is again dissolved in an IL, modified and then transformed into the desired stationary phase.

Focusing on an entirely different aspect, ILs for whole-cell transformations, Fan *et al.* [8] review recent developments in the field and highlight how and why specifically developed ILs can add value to such processes. Moreover, the authors also show that many of the underlying mechanisms are not well understood, and note which questions will need to be addressed in the future. As such, the article is a very good starting point for anyone wanting to delve deeper into the subject.

A further group of articles in this special issue is devoted to extraction, ranging from extraction of light metals and rare elements, such as the platinum group metals [9], and rare earths [10] to parabens [11] and pesticides [12]. While different in their individual approaches, the overarching theme of these articles is the exploitation of the unique properties of ILs, specifically the favorable interaction of very different chemical species (metal ions and organic molecules with very different hydrohilicities), with IL cations and anions which in turn enable the specific extraction to take place. These examples clearly illustrate that ILs also have an “indirect” materials aspect in the sense that ILs can provide new access strategies towards valuable components for materials synthesis, such as the rare earths.

Finally, there are a few articles that have a “synthesis” focus. Three articles describe IL catalysis as a tool for organic chemistry. He *et al.* [13], show the first examples of Mannich reactions catalyzed by geminal ILs. Using the same basic motive, a sulfobetaine, Wang *et al.* [14] demonstrate efficient IL-catalyzed Aldol condensation. Hu *et al.* [15] used a choline hydroxide IL for the synthesis of 4H-benzo[*b*]pyrans. Last, but not least, returning to the subject of the ILMAT 2013—materials chemistry with ionic liquids—Ibrahim *et al.* [16] demonstrate that ILs can also be used to enhance the solubility of CO_2_ in aqueous environments; the increased CO_2_ concentration can be exploited for the controlled carbonation of Ca(OH)_2_ slurries, a process that has clear implications for large scale use.

Overall, the diversity of topics described in the articles contained within the special issue clearly shows that ILs do add value to various aspects of chemistry. The current special issue especially showcases examples from materials chemistry, carbohydrate chemistry, extraction and organic synthesis. As the editors of the special issue, we now invite you to browse the special issue and to actively engage in the field of ionic liquid-based (materials) chemistry. Finally, we hope to see many of you at the ILMAT 2015 in Potsdam.
